# No evidence for parental age effects on offspring leukocyte telomere length in free-living Soay sheep

**DOI:** 10.1038/s41598-017-09861-3

**Published:** 2017-08-30

**Authors:** H. Froy, E. J. Bird, R. V. Wilbourn, J. Fairlie, S. L. Underwood, E. Salvo-Chirnside, J. G. Pilkington, C. Bérénos, J. M. Pemberton, D. H. Nussey

**Affiliations:** 1 Institute of Evolutionary Biology, University of Edinburgh, Edinburgh, EH9 3FL UK; 2 SynthSys, University of Edinburgh, Edinburgh, EH9 3BF UK

## Abstract

In humans, the effect of paternal age at conception (PAC) on offspring leukocyte telomere length (LTL) is well established, with older fathers thought to pass on longer telomeres to their offspring in their sperm. Few studies have looked for PAC effects in other species, but it has been hypothesised that the effect will be exacerbated in polygamous species with higher levels of sperm competition and production. We test for maternal (MAC) and paternal age at conception effects on offspring LTL in Soay sheep, a primitive breed experiencing strong sperm competition. We use qPCR to measure relative telomere length in 389 blood samples (n = 318 individuals) collected from an unmanaged population of sheep on St Kilda, where individual age and parentage are known. We find no evidence that either MAC or PAC are associated with LTL in offspring across the age range, or when considering only young lambs (n = 164). This is the first study to test for parental age effects on offspring LTL in a wild mammal population, and the results contrast with the findings of numerous human studies that find a PAC effect, as well as predictions of a stronger PAC effect in polygamous species.

## Introduction

Telomeres are non-coding repeat sequences of DNA (TTAGGG) at the ends of eukaryotic chromosomes. Along with proteins, they cap chromosome ends to allow DNA repair machinery to distinguish them from breaks in DNA and prevent coding DNA from being degraded during cell replication^1, 2^. Telomere shortening occurs as a result of normal cell division and damage from reactive oxygen species, but can be maintained through telomerase activity^2^. When telomeres reach a critically short length cells undergo cell death or replicative senescence, and so telomere dynamics are closely linked to cell function and cellular ageing^1, 3^. In humans, average leukocyte telomere length (LTL) has been shown to be highly heritable, to shorten with age and with past experience of chronic stress, and to be associated with greater risk of subsequent morbidity and mortality^4–7^. Emerging evidence from diverse species of birds and reptiles suggests that findings from human studies may generalise to other vertebrate species. In particular, there is mounting evidence that erythrocyte telomere length across diverse avian species may be heritable and is associated with prior stress and subsequent survival in both laboratory and wild populations^8–12^. Understanding the causes of variation in the average blood cell telomere length of an individual organism has become an important question within both human epidemiology and evolutionary ecology.

One particularly robust and repeatable predictor of LTL in humans is paternal age at conception (PAC). Numerous studies of different human populations, summarised in Table 1, have documented a positive association between offspring LTL, typically measured in adulthood, and PAC. One particular challenge in human studies is separating the effects of PAC and maternal age at conception (MAC) on LTL, as PAC and MAC are typically very highly correlated in human populations (see Table 1). Although MAC is often positively correlated with offspring telomere length when considered alone, the majority of studies have found that only PAC remains as a significant predictor of offspring LTL when both parents’ ages are fitted in statistical models (Table 1). This has been interpreted as suggesting that the positive association between MAC and offspring LTL is largely the result of the close correlation between MAC and PAC, rather than any underlying biological effect of maternal age^13, 14^. There is evidence that older men produce sperm with longer telomeres than younger men, and it has been hypothesised that inheritance of these longer telomeres could explain the widely observed PAC effect on offspring LTL^14, 15^. In support of this, a recent study found a positive effect of PAC on telomere length in new-borns, suggesting that the effect is likely to be present at fertilisation^16^. Two non-exclusive theories have been put forward to explain why older men should produce sperm with long telomeres. The first is that germ stem cells (GSCs) with longer telomeres possess some property that makes them better able to withstand the repeated cell divisions and survive, and therefore come to predominate in the sperm stem cell pool with age^14, 17^. The second theory is that, due to constant sperm production and cell division in the male germline in humans, telomerase is highly active to compensate for telomere loss. Rather than simply maintaining telomere length, GSC telomerase expression “overshoots” and elongates telomeres in these cells^14, 18^.

These mechanistic hypotheses suggest that the PAC effect in humans is related to the need for constant sperm production in the male germline, meaning it could generalise to other mammal species. A recent study in 40 captive chimpanzees (*Pan troglodytes*) found that offspring with older fathers had longer telomeres as adults, and this PAC effect was in fact much steeper than in humans^19^. Chimpanzees have a more promiscuous mating system than humans, which has selected for much larger testes and higher rates of sperm production^20^. It was suggested that this increased sperm competition could exacerbate the drivers of the PAC effect observed in humans, since they are associated with the proliferative process of sperm production^19^. We would therefore expect the PAC effect to be strongest in polygamous promiscuous species, in which levels of sperm competition and rates of sperm production are often far higher than in humans. However, tests of the generality of PAC effects on blood cell telomere length across species are currently very limited (Table 1). In non-mammalian vertebrates, a handful of studies have tested for PAC effects and found patterns inconsistent with those observed in humans and chimpanzees. In the sand lizard (*Lacerta agilis*), older fathers were found to have sons with shorter telomeres, which is the opposite of what is reported in humans^21^. In European shags (*Phalacrocorax aristotelis*), parental age was not associated with offspring telomere length at hatching, but chicks raised by older parents had greater telomere loss from hatching to 1 month old than those reared by younger parents^22^. This suggests the effect was linked to parental provisioning of the neonatal offspring rather than inheritance of shorter telomeres from older parents. However, MAC and PAC were so strongly correlated in this system that their effects could not be separated^22^. In another species of bird, the great reed warbler (*Acrocephalus arundinaceus*), a positive relationship was found between MAC, but not PAC, and offspring telomere length^8^.

Here, we investigate the effect of MAC and PAC on offspring leukocyte telomere length (LTL) in a free-living population of Soay sheep (*Ovis aries*) living on the remote St Kilda archipelago. Our data come from sheep resident to the Village Bay area of the main island of Hirta. This population has been the subject of regular individual-based monitoring since 1985, and the parents of most lambs born in the study population have been determined by either observational or molecular methods^23, 24^. The Soay sheep has a highly polygynous mating system where males compete for access to females during the annual autumn rut^24^. Since females mate multiply during their receptive oestrus period, sperm competition is also apparent in this species. This results in strong selection on sperm production, which is evident in the very high testes to body mass ratio seen in male Soay sheep^25, 26^ (Soay sheep 0.58%^27^ compared to humans 0.06% and chimpanzees 0.27%^28^). If the PAC effect observed in humans is associated with constant sperm production by GSCs driving telomere lengthening in sperm with age, then we would expect the effect to generalise to other mammals and potentially be stronger in highly promiscuous species like sheep. Whilst maternal age effects on offspring phenotypes are widely observed in this population^29, 30^, paternal age effects of any kind have yet to be documented. We have previously developed and validated a qPCR-based approach for measuring LTL in Soay sheep and have shown that LTL is positively associated with survival in females in this population^31^. More recently, we measured LTL in a cross-sectional sample of animals, spanning both sexes and the full age range, and found an age-dependent sex difference in LTL in this system which mirrors that commonly observed in humans^32^. In this study, we utilise the unusually detailed information on parentage available in this system to provide the first test for MAC and PAC effects on offspring LTL in a wild mammal.

## Results

**Figure 1:**
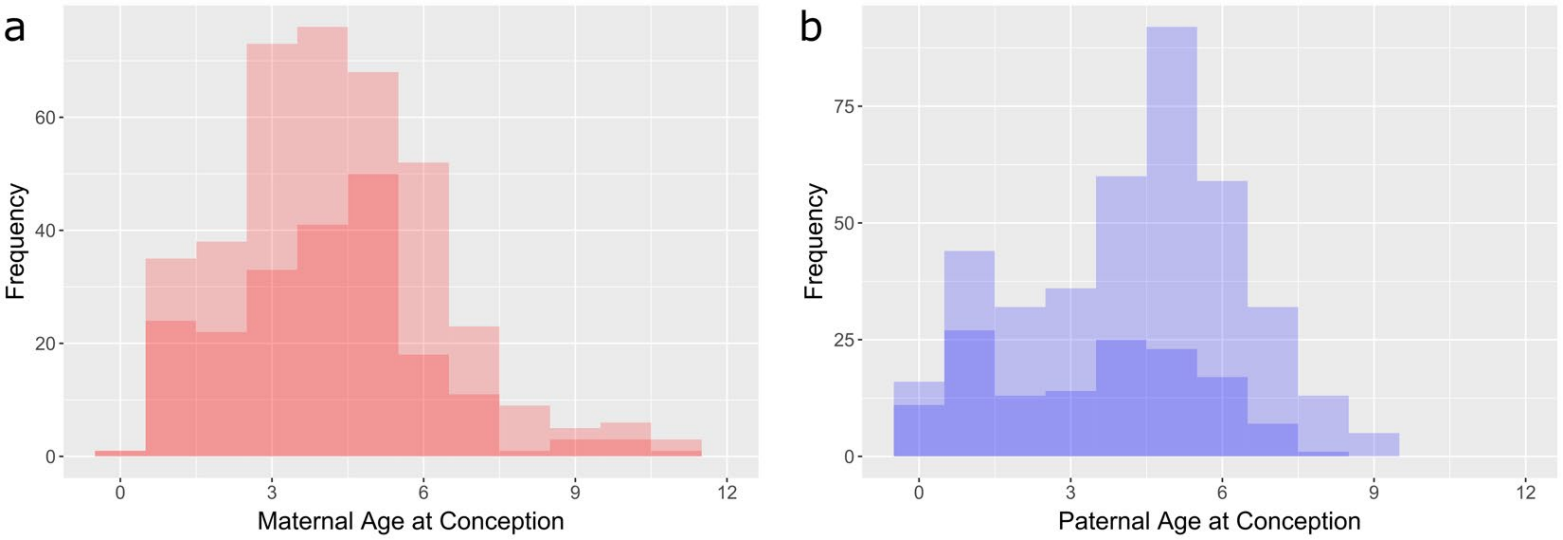
Histograms showing frequency of (**a**) maternal and (**b**) paternal ages at conception amongst offspring with telomere length measurements used in this study. The darker underlying histogram shows the frequency of mean (**a**) maternal and (**b**) paternal age at conception of individual parents who had more than one offspring included in the study.

**Figure 2:**
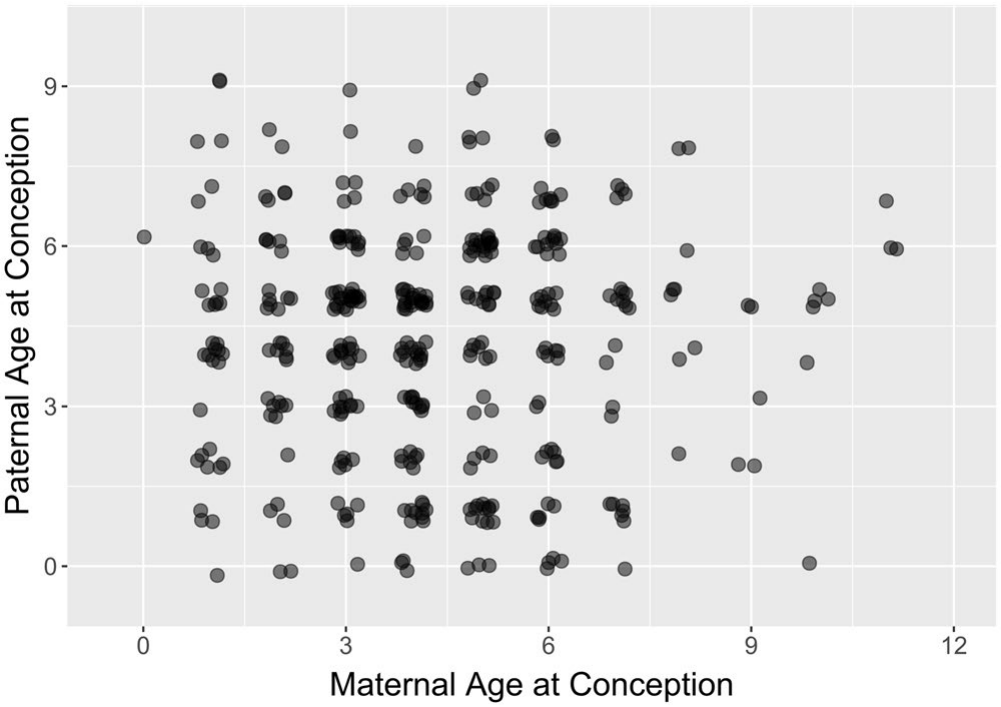
Scatterplot illustrating the absence of any correlation between paternal and maternal age at conception for individuals measured in this study. Parental ages are year integers, but points are jittered to show the amount of data at each combination of ages.

**Figure 3:**
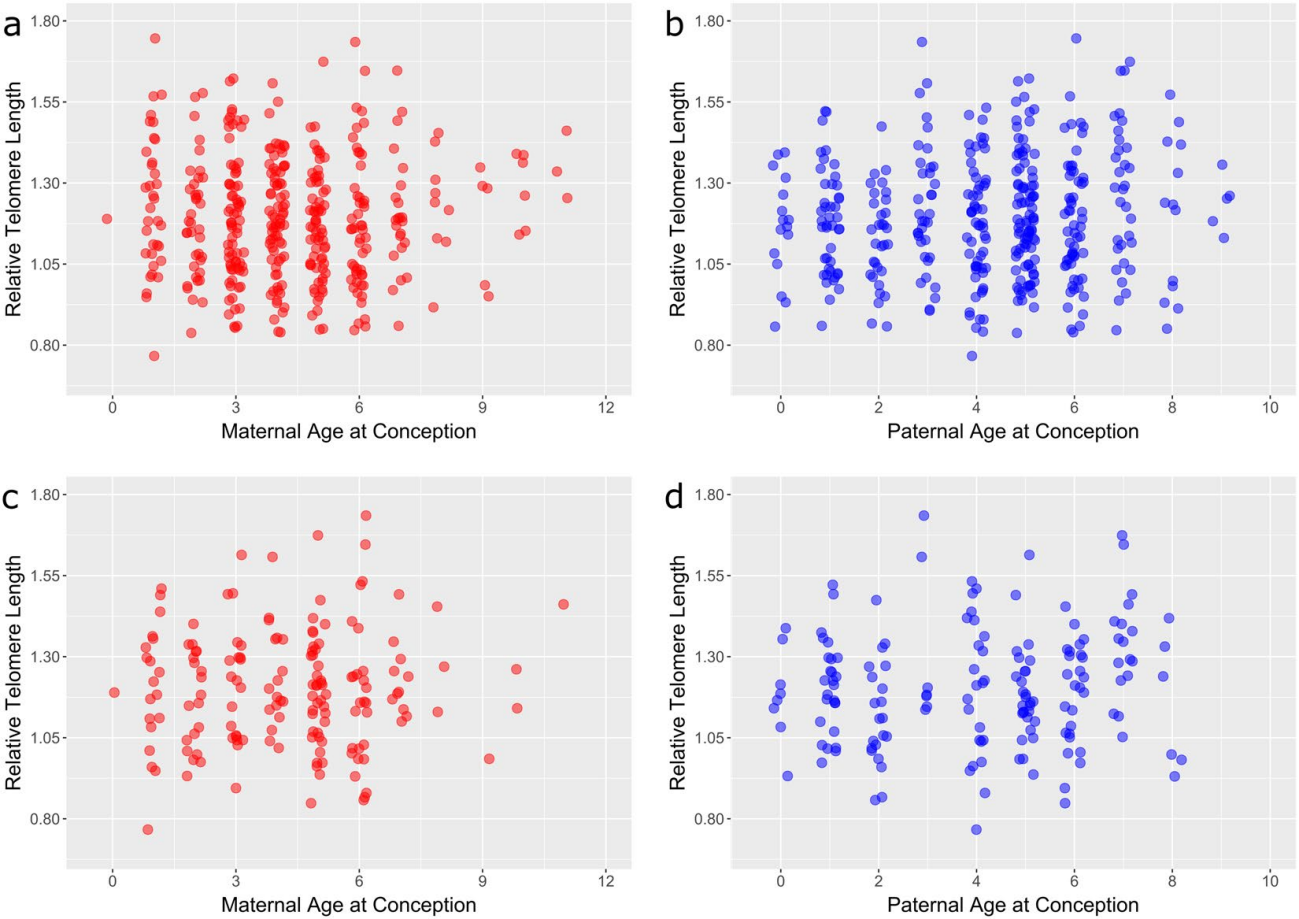
No significant associations between offspring relative telomere length and either (**a**,**c**) maternal or (**b**,**d**) paternal age at conception in free-living sheep. Scatterplots of telomere length for all individuals in the sample (**a**,**b**) and for lambs aged around four months only (**c**,**d**).

There were 389 measurements of relative telomere length (RTL) from 318 individual sheep where maternal (MAC) and paternal age at conception (PAC) were known. The distributions of MAC and PAC in our dataset spanned the range of reproductive ages observed in the population (Fig. 1), and MAC and PAC were not correlated (Pearson’s correlation coefficient = 0.007, p = 0.895; Fig. 2). MAC did not explain significant variation in RTL when added to a model including year, age, sex and the interaction between age and sex (linear MAC effect: χ^2^_(d.f. = 1)_ = 0.019, p = 0.891; quadratic effect: χ^2^_(2)_ = 5.539, p = 0.063; Fig. 3a). Similarly, PAC did not explain significant variation in RTL when added to the same model (linear PAC effect: χ^2^_(1)_ = 2.675, p = 0.102; quadratic effect: χ^2^_(2)_ = 2.969, p = 0.227; Fig. 3b). The proportion of variance in the random effects component of our models explained by mothers and fathers was 16% and 2%, respectively (see Table S1 for model summaries). When considering only RTL measured in lambs aged approximately four months (n = 164), neither MAC nor PAC explained significant variation in RTL (linear effects: MAC χ^2^_(1)_ = 0.601, p = 0.438; PAC χ^2^_(1)_ = 1.397, p = 0.237; quadratic effects: MAC χ^2^_(2)_ = 0.803, p = 0.669; PAC χ^2^_(2)_ = 3.246, p = 0.197; Fig. 3c,d) (see Table S2 for model summaries).

## Discussion

In this study, we find no evidence that offspring telomere length is related to maternal or paternal age at conception in wild Soay sheep. The effect of PAC on offspring telomere length is well established in humans, with large-scale studies across a wide range of offspring ages consistently finding that older fathers have offspring with longer telomeres (Table 1). Studies in non-human mammals are rare, but a similar finding has recently been reported in captive chimpanzees^19^. Our study is the first to describe effects of parental age on offspring telomere length in a wild mammal population, and we find no evidence that increasing paternal age is associated with longer telomeres in either adult or juvenile offspring (Fig. 3).

**Table 1:** Studies testing for effects of paternal (PAC) and maternal (MAC) age at conception on offspring telomere length. Tissue types: white blood cells (WBC); mononuclear cells (MNC); red blood cells (RBC).

Species	Class	Tissue	Method	Offspring sample size	Offspring age range	PAC effect alone	MAC effect alone	PAC/MAC effect together	MAC/PAC correlation coef	Reference
*Homo sapiens*	Mammalia	WBC	TRF	2177	31–86 years	Positive	Positive	PAC positive No MAC effect	0.83	50
*Homo sapiens*	Mammalia	WBC	qPCR	19713	28–59 years	Positive	Positive	PAC positive No MAC effect	—	5
*Homo sapiens*	Mammalia	WBC	TRF	2281	35–55 years	Positive	Positive	PAC positive No MAC effect	0.84	13
*Homo sapiens*	Mammalia	WBC	qPCR	2023	21–23 years	Positive	Not reported	Not reported	—	51
*Homo sapiens*	Mammalia	WBC	TRF	490	Newborn	Positive	None	Not reported	0.8	16
*Homo sapiens*	Mammalia	WBC	TRF	889 twin pairs	18–76 years	Positive	Positive	Not reported	0.72–0.83	17
*Homo sapiens*	Mammalia	WBC	qPCR	4250	42–69 years	Positive	None	PAC positive MAC negative	0.77	52
*Homo sapiens*	Mammalia	WBC	TRF	125	30–80 years	Positive	None	Not reported	—	53
*Homo sapiens*	Mammalia	WBC	TRF	3365	18–94 years	Positive	Not reported	PAC positive No MAC effect	0.72–0.85	14
*Homo sapiens*	Mammalia	WBC	qPCR	907	18–92 years	Positive	Positive	Not reported	—	54
*Homo sapiens*	Mammalia	WBC	qPCR	73	20–56 years	Variable^a^	Not reported	Not reported	—	55
*Homo sapiens*	Mammalia	MNC	qPCR	49	32–42 years	None	Not reported	Not reported	—	56
*Homo sapiens*	Mammalia	WBC	qPCR	98 PAC, 129 MAC	0–102 years	None	None	Not reported	—	40
*Homo sapiens*	Mammalia	WBC	qPCR	96	4–5 years	Positive	Not reported	PAC positive No MAC effect	—	57
*Homo sapiens*	Mammalia	WBC	qPCR	144	—	Positive	Not reported	Not reported	—	19
*Pan troglodytes*	Mammalia	WBC	qPCR	40	6–57 years	Positive	Not reported	Not reported	—	19
*Acrocephalus arundinaceus*	Aves	RBC	qPCR	154 PAC	8–10 days	Not reported	Not reported	No PAC effect MAC positive	—	58
*Acrocephalus arundinaceus*	Aves	RBC	qPCR	207	8–10 days	Not reported	Variable^b^	Not reported	—	8
*Phalacrocorax aristotelis*	Aves	RBC	qPCR	204 PAC	0–35 days	Negative^c^	Negative^c^	Not reported	0.71–0.73	22
*Lacerta agilis*	Reptilia	RBC	TRF	12	2–6 years	Negative^d^	Not reported	Not reported	—	21

^a^No relationship in control group, positive in male offspring and negative in female offspring (Schizophrenic group).

^b^Positive in uninfected mothers, negative in malarial infected mothers.

^c^Relationship observed at fledgling but not hatchling stage.

^d^Negative in male offspring, data for female offspring not available.

The PAC effect in humans is hypothesised to be driven by the proliferative process of sperm production, and it has been suggested that polygamous species experiencing high levels of sperm competition should show a strong PAC effect^19^. In wild Soay sheep on St Kilda, sexual selection is strong and males face strong pre-copulatory competition for access to females, whilst there is also strong post-copulatory sperm competition since females can mate multiply during their receptive oestrus period^26^. Paternity share is thought to be proportional to the number of sperm inseminated by each competitor. This ‘raffle mechanism’ of sperm competition means there is strong selection on sperm production, which appears to have selected for an exceptionally high testes to body mass ratio in Soay sheep^25–27^. We predicted that high rates of sperm production in the sheep would exacerbate the mechanisms thought to drive the PAC effect. Firstly, if rates of sperm production are higher, we may expect telomerase to be more active in the male germline in order to counteract telomere shortening caused by successive rounds of cell division. Increased rounds of replication and telomerase “overshoot” may therefore result in older male sheep producing sperm with progressively longer telomeres, which they pass on to their offspring. Secondly, the selection hypothesis suggests that germ stem cells (GSCs) with longer telomeres are better suited to survive in an ageing environment and therefore come to predominate in the gonads of older males. We may expect this selection against GSCs with shorter telomeres to be exacerbated if sperm production is more rapid, resulting in a stronger increase in average sperm telomere length with age and a more pronounced PAC effect. These predictions are supported by recent findings in captive chimpanzees, which are promiscuous and show a strong effect of PAC on offspring telomere length^19^.

There are a number of plausible explanations for the surprising lack of evidence for a PAC effect on LTL in Soay sheep. Given the strong selection on sperm production in this promiscuous species, it may be that telomerase activity in the germline is more tightly regulated, and therefore it does not “overshoot” as in humans. Alternatively, telomerase expression may be lower in the sheep germline. Telomerase expression varies hugely among tissue types, but also among species. Among mammals, telomerase expression appears to be suppressed in somatic cells in larger species, although pigs and tigers are noted exceptions to this rule^33, 34^. However, a detailed understanding of how tissues vary in telomerase expression among mammals is lacking; our current understanding comes mostly from *in vitro* studies of cultured fibroblasts from different species^35^. Additionally, if telomere length relates to the persistence and survival of GSCs, and higher levels of sperm competition in the sheep results in stronger selection on the male germline, it is possible that this will reduce the variability of telomere length within the male GSC pool. If the composition of telomere lengths within the germline is more consistent, selective loss of GSCs with age will have less impact on the average telomere length in sperm, and thus offspring may not inherit longer telomere lengths from older fathers on average.

It is also important to note that Soay sheep, although reasonably long-lived (maximum recorded lifespan for males 10 years, females 16 years), have a much lower life expectancy than both humans and chimpanzees. It could be that male sheep simply do not live long enough for the lengthening of telomeres in sperm to become apparent. For example, it may be that the majority of GSCs persist throughout the relatively short lifespan of a male sheep, and therefore subsampling of GSCs with longer telomeres does not occur. Though we detect reproductive senescence in the longer-lived female Soay sheep, we do not observe a marked decline in male reproductive success with age^29^. This contrasts with findings of increased infertility with age in humans^36^, and raises the possibility that male sheep may be able to maintain healthy sperm production for the majority of their lives. This may in part be due to the seasonal pattern of reproduction in sheep^37^. Sperm production does continue year round in domestic sheep^38^, and the very late birth dates of some lambs on St Kilda suggests that sperm production in Soays continues well into the spring at least. However, testicular circumference increases dramatically around the annual autumn rut in Soay rams^39^. If the rate of sperm production is lower throughout the rest of the year, it is possible that the average rate of sperm production over the year is not very high, despite the high rates of sperm competition and polygamous mating system.

In our study of Soay sheep, we were able to effectively separate MAC and PAC since these are not correlated in our system (Fig. 2). We were also able to restrict our analyses to offspring of only four month olds, which should reduce environmental influences on telomere length. However, we still failed to detect a maternal or paternal age at conception effect (Fig. 3c,d). We also found a low repeatability of telomere length in offspring from the same parents, suggesting heritability of LTL is likely to be relatively low in Soay sheep. This may be because Soay sheep live in a much more variable environment than humans (particularly since participants in telomere studies may be a relatively homogenous subset of the population). They experience dramatic variation in food availability, and sometimes prolonged periods of food shortage, very high parasite burdens, intense male-male competition, and females have a high reproductive output. Individuals also show low repeatability in telomere length over time when compared to human studies^31^. It may be that these influences on telomere length obscure the pattern of genetic inheritance as well as maternal and paternal age at conception effects. We conducted a simulation-based power analysis, accounting for the structure of our model and data, and found that we have 80% power to detect a PAC effect of 0.012 or greater (see Fig. S1). Given that telomere length measured by qPCR is expressed as a relative value, and that the range of paternal ages varies widely among species, comparing PAC effect sizes across studies is not straightforward. However, multiplying the effect size of 0.012 by the standard deviation of PAC (SD_PAC_ = 2.121) over the standard deviation of RTL (SD_RTL_ = 0.185) in our dataset indicates this is equivalent to a correlation coefficient of r = 0.140. The PAC effect reported in the recent chimpanzee study^19^ was nearly three times this size at r = 0.378, and the PAC effect reported in humans in the same study was of a comparable size at r = 0.136. Correlation coefficients of a similar size have also been reported in other human studies (r = 0.127^13^; r = 0.160^40^). This suggests that if a PAC effect equivalent to that reported in humans exists in Soay sheep, we have sufficient power to detect it with our dataset, and we have even greater power to detect a larger PAC effect as predicted by the polygamous mating system (100% power to detect a PAC effect equivalent to that reported in chimpanzees). Though our sample sizes are much larger than any other non-human PAC study (Table 1), if the effect of PAC is very small and the environment highly variable, we may need even larger samples sizes to detect weak, positive PAC effects in wild mammal populations.

This is the first study to examine the influence of maternal and paternal age at conception on offspring telomere length in a wild mammal population. In stark contrast to many human studies, which find that older fathers have offspring with longer telomeres, and against predictions of a stronger paternal age effect in polygynous promiscuous species, we find no evidence for MAC or PAC effects in Soay sheep. This adds to a small but growing body of literature that suggests that the PAC effect detected in humans is not universal (Table 1). Further studies are needed to disentangle within-individual effects of PAC on offspring telomere length, and to better understand how and why PAC effects vary across species.

## Methods

### Study system & sample collection

We used data from a long-term study of Soay sheep on St Kilda. This primitive breed has inhabited islands within the remote St Kilda archipelago for thousands of years. Since 1985, the population resident within the Village Bay area of the main island of Hirta has been subject to individual-based study^24^. Lambs are individually marked within a few days of birth in spring, and in August each year 50–60% of the resident population are caught and blood sampled. Blood is collected into lithium heparin vacuettes and spun within 24 hours to separate the plasma and buffy coat fractions. Buffy coat samples, comprising mainly white blood cells, are stored at −20 °C until used to assay leukocyte telomere length. This study used samples from animals caught in August 2014 (83 males and 177 females) and August 2015 (66 males and 175 females). All data collection was approved by the UK Home Office and carried out in accordance with the relevant guidelines (Home Office Project License PPL 60/3547).

### Pedigree reconstruction

Parentage was inferred by genetic methods, except for some maternal links inferred by observation^23, 41^. Two methods were used for molecular parentage assignments. The first was a cohort-based method using 310 polymorphic and unlinked SNP markers in the R package *MasterBayes* ^42^. For each cohort of lambs, the candidate parent list was restricted to individuals born at least a year prior to the cohort of interest, and to individuals that were known to be alive during the previous three years. Candidate parents were discarded if showing more than 8 mismatches, and the chain was subsequently run for 13000 iterations, with a burn-in of 3000 iterations and a thinning interval of 10 iterations. Parental links were inferred with 100% confidence. The second parentage inference method was multi-generational pedigree reconstruction in the R package *Sequoia* ^43^, using 310 unlinked SNP markers. This likelihood-based approach infers not only parent-offspring relationships, but also sibs and second degree relatives. Across the whole pedigree, *Sequoia* identified 137 and 117 dams and sires respectively which were not assigned using *MasterBayes*. Conversely, 61 and 141 dams and sires were assigned using *MasterBayes* but not in *Sequoia*. Where both methods inferred a parent, different assignments were made for one maternal and one paternal link only and the *MasterBayes* assignments were used. This enabled the construction of a pedigree with a maximum depth of 11 generations and consisting of 8221 individuals, of which 7771 were non-founders and a total of 7169 maternities and 5527 paternities were assigned.

### Telomere length measurement

Genomic DNA was extracted from buffy coat using the Qiagen DNeasy Blood and Tissue Kit (Cat# 69581, Manchester, UK), following the manufacturer’s guidelines for animal blood with the following minor modifications. Initially, 50 µl of buffy coat was mixed thoroughly with 300 µl Qiagen Red Blood Cell (RBC) lysis solution (Cat# 158902, Manchester, UK) and then centrifuged for 30 s at 14000 × g to produce a white blood cell (WBC) pellet. The supernatant was then discarded leaving approximately 10 µl residual liquid. 100 µl PBS was added to the sample and the WBC pellet re-suspended by vortexing. This step removes all remaining red blood cells. The volume of proteinase K added was increased from 20 µl to 30 µl to ensure complete WBC digestion, and the duration of incubation with buffer AL at 56 °C was increased from 10 min to 1 hour, vortexing the sample at 30 min mid-incubation, to optimise WBC lysis. Following DNA extraction and elution in buffer AE (10 mM TrisCl, 0.5 EDTA, pH 9.0), a strict quality control protocol was implemented to determine DNA quality and integrity. First, each sample was individually tested for DNA yield and purity using a Nanodrop ND-1000 9 spectrophotometer (Thermo Scientific, Wilmington DE, USA). Samples yielding <20 ng/µl were immediately rejected. Samples yielding ≥20 ng/µl were checked for DNA purity; acceptable ranges for absorption were 1.7–2.0 for 260/280 nm ratio and 1.8–2.2 for 260/230 nm ratio. Acceptable samples were then diluted to 10 ng/µl and their DNA integrity assessed by running 20 µl (200 ng total DNA) on a 0.5% agarose gel. Samples were scored for integrity on a scale of 1 to 5 by visual examination of their DNA crowns, with samples scoring higher than 2 being excluded from further analyses (see^44^ for details). Samples which failed one or more of the above QC measures were re-extracted and if they failed QC a second time they were excluded from the study.

Relative leukocyte telomere length (RTL) was measured using real-time quantitative PCR (qPCR^45^), using protocols we have previously developed and validated in sheep blood samples^31, 44^. The qPCR method estimates the total amount of telomeric sequence present in a sample relative to the amount of a non-variable copy number reference gene. In this study we used the beta-2-microglobulin (B2M) as our reference gene, using primers supplied by Primer Design (Cat# HK-SY-Sh-900, Southampton, UK). For telomeric amplification Tel 1b (5′-CGG TTT GTT TGG GTT TGG GTT TGG GTT TGG GTT TGG GTT-3′) and Tel 2b (5′-GGC TTG CCT TAC CCT TAC CCT TAC CCT TAC CCT TAC CCT-3′) primers were used^7^. Telomere primers were manufactured, HPLC purified and supplied by Integrated DNA Technologies (IDT, Glasgow, UK). Telomere and reference gene reactions were run in separate wells of the same qPCR plate at a concentration of 300 nM and 900 nM respectively. Samples were diluted to 1 ng/µl with buffer AE just prior to qPCR analysis. Each reaction was prepared using 5 µl of LightCycler 480SYBR Green I Master Mix (Cat # 04887352001, Roche, West Sussex, UK), 0.5 µl B2M primer or 0.6 µl Tel primer, and 1 ng of sample DNA in a total reaction volume of 10 µl. 384 well plates were loaded with sample DNA and master mix using an automated liquid handling robot (Freedom Evo-2 150; Tecan).

Each plate included two calibrator samples (1 ng/µl) to account for plate to plate variation, and a non-template control (NTC) consisting of nuclease free water. The calibrator sample was extracted from a large quantity of buffy coat prepared from blood supplied from a single domestic sheep (Cat# SHP-BUFCT-LIHP, Sera Laboratories International LTD, West Sussex, UK). We carried out a large number of extractions from this sample, applied the same quality control as above and then pooled the extracts and aliquoted them for subsequent use. Samples, calibrators and NTCs were all run in triplicate. All qPCRs were performed using a Roche LC480 instrument using the following reaction protocol; 10 min at 95 °C (enzyme activation), followed by 50 cycles of 15 s at 95 °C (denaturation) and 30 s at 58 °C (primer annealing), then 30 s at 72 °C (signal acquisition). Melting curve protocol was 1 min at 95 °C, followed by 30 s at 58 °C, then 0.11 °C/s to 95 °C followed by 10 s at 40 °C.

We used the LinRegPCR software package (version 2016.0^46^) to correct our amplification curves for baseline fluorescence, and to calculate well-specific reaction efficiencies and Cq values. A constant fluorescence threshold was set within the window of linearity for each amplicon group, calculated using the average Cq across the first 6 plates. The threshold values used were 0.193 and 0.222, and the average efficiency across all plates were 1.88 and 1.91 for the B2M and telomere amplicon groups, respectively. Samples were excluded from further analysis if the coefficient of variation (CV) across triplicate Cq values for either amplicon was >5%, or if at least one of their triplicate reactions had an efficiency that was 5% higher or lower than the mean efficiency across all wells on that plate for the respective amplicon. The technical repeatability of RTL (or intraclass correlation coefficient) was estimated to be 0.87 for a previous study in our lab, where RTL was estimated for 48 Soay sheep samples using the same protocols as this study, run in quadruplicate^44^. Overall, ten samples were excluded based on quality control failure at either extraction or qPCR stages, leaving 491 samples available for use in further analyses.

Relative LTL for each sample was calculated, following^47^, using average reaction efficiencies for each plate and Cq for each sample determined by LinRegPCR as follows:

$${\rm{RTL}}=({{{\rm{E}}}_{{\rm{TEL}}}}^{({\rm{CqTEL}}[{\rm{Calibrator}}]\mbox{--}{\rm{CqTEL}}[{\rm{Sample}}])})/({{{\rm{E}}}_{{\rm{B}}2{\rm{M}}}}^{({\rm{CqB2M}}[{\rm{Calibrator}}]\mbox{--}{\rm{CqB}}2{\rm{M}}[{\rm{Sample}}])})$$

where E_TEL_ and E_B2M_ are the mean reaction efficiencies for the respective amplicon group across all samples on a given plate; CqTEL[Calibrator] and CqB2M[Calibrator] are the average Cqs for the relevant amplicon across all calibrator samples on the plate; and CqTEL[Sample] and CqB2M[Sample] are the average of the triplicate Cqs for the sample for each amplicon.

### Statistical analyses

Of the 491 samples available, there were 389 RTL measurements from 318 individual sheep where maternal (MAC) and paternal age at conception (PAC) were known (134 and 140 females and 66 and 49 males in 2014 and 2015, respectively). There were 208 unique mothers and 138 unique fathers in the data set. There were three pairs of full-sibs, 110 maternal half-sibs and 180 paternal half sibs. All analyses were conducted in the statistical program R (v.3.2.2)^48^.

Variation in RTL was modelled using linear mixed effect models (LMMs) in the R package *lme4* ^49^. Our base model included the following fixed effects: sample year as a 2-level factor; age as a covariate; sex as a 2-level factor; and the interaction between age and sex^32^. Maternal identity, paternal identity and qPCR plate (samples were run on 9 different plates) were included as random intercept terms to account for non-independence of observations. We examined the effect of MAC and PAC on RTL by adding these terms to our base model both as linear and quadratic terms. The significance of these terms was assessed using likelihood ratio tests. In all cases, models comparing different fixed effect structures were estimated with maximum likelihood (ML). To check that any effects of MAC and PAC on RTL were not masked by including adults in our analyses, we repeated the above restricting our dataset to lambs. There were 164 measurements of RTL in lambs, who were all sampled at approximately four months old (n = 84 females; n = 80 males from 119 unique mothers and 69 fathers). We used the same approach as above, though age and its interaction with sex were not included since all individuals were the same age.

### Data availability

The data used in this study are available in the Supplementary materials.

## Electronic supplementary material

Supplementary materials

Dataset 1
