# Supplementary material for: No evidence for parental age effects on offspring leukocyte telomere length in free-living Soay sheep
Source: Sci Rep. 2017 Aug 30;7:9991. doi: 10.1038/s41598-017-09861-3 (PMC5577307; doi:10.1038/s41598-017-09861-3)
Supplement: Supplementary file 1 — Supplementary materials [file 41598_2017_9861_MOESM1_ESM.doc]

**Supplementary Materials for: No evidence for parental age effects on offspring leukocyte telomere length in free-living Soay sheep**

Froy, H., Bird, E.J., Wilbourn, R.V., Fairlie, J., Underwood, S.L., Salvo-Chirnside, E., Pilkington, J.G., Bérénos, C., Pemberton, J.M., & Nussey, D.H.

**Table S1. Fixed and random effect estimates from linear mixed effects models of relative telomere length (RTL) in Soay sheep (n = 389 observations), testing effects of a) MAC and b) PAC on RTL. p values are from likelihood ratio tests comparing models with and without each term (and any associated interactions). Effects of fixed factors are shown relative to a reference level (Sex Females; Year 2014).**

|  | **Random effects** | **Variance** |  | **Fixed effects** | **Estimate** | **Std error** | **p value** |
| --- | --- | --- | --- | --- | --- | --- | --- |
| **a)** | Mum ID | 0.005 |  | Age | -0.001 | 0.003 | 0.199 |
|  | Dad ID | 0.001 |  | Sex (Male) | 0.008 | 0.024 | 0.260 |
|  | Plate | 0.004 |  | Year (2015) | -0.011 | 0.017 | 0.517 |
|  | Residual | 0.025 |  | MAC | 0.001 | 0.005 | 0.891 |
|  |  |  |  | Age : Sex (Male) | -0.014 | 0.009 | 0.116 |
| **b)** | Mum ID | 0.005 |  | Age | 0.000 | 0.003 | 0.191 |
|  | Dad ID | 0.001 |  | Sex (Male) | 0.012 | 0.024 | 0.240 |
|  | Plate | 0.004 |  | Year (2015) | -0.014 | 0.017 | 0.409 |
|  | Residual | 0.025 |  | PAC | 0.007 | 0.004 | 0.102 |
|  |  |  |  | Age : Sex (Male) | -0.014 | 0.009 | 0.098 |

**Table S2.** Fixed and random effect estimates from linear mixed effects models of RTL in Soay sheep lambs at four months (n = 164 observations), testing effects of a) MAC and b) PAC on RTL. p values are from likelihood ratio tests comparing models with and without each term. Effects of fixed factors are shown relative to a reference level (Females for Sex; 2014 for Year).

|  | **Random effects** | **Variance** |  | **Fixed effects** | **Estimate** | **Std error** | **p value** |
| --- | --- | --- | --- | --- | --- | --- | --- |
| **a)** | Mum ID | 0.005 |  | Sex (Male) | 0.008 | 0.024 | 0.700 |
|  | Dad ID | 0.002 |  | Year (2015) | -0.011 | 0.017 | 0.172 |
|  | Plate | 0.003 |  | MAC | 0.001 | 0.005 | 0.438 |
|  | Residual | 0.022 |  |  |  |  |  |
| **b)** | Mum ID | 0.004 |  | Sex (Male) | 0.012 | 0.024 | 0.650 |
|  | Dad ID | 0.001 |  | Year (2015) | -0.014 | 0.017 | 0.169 |
|  | Plate | 0.003 |  | PAC | 0.007 | 0.004 | 0.237 |
|  | Residual | 0.023 |  |  |  |  |  |

**Figure S1.** The power we have to detect PAC effects of varying sizes with our dataset. Results are from a simulation-based power analysis for linear mixed effects models conducted using R package *simr*^1^. Points and black lines show mean power and 95% confidence intervals each estimated from 500 simulations. Grey lines show that we have 80% power to detect a PAC effect of 0.012 or larger, accounting for the specification of our model and the structure of our data. This is equivalent to a correlation coefficient of r = 0.14 (see Discussion in main text).


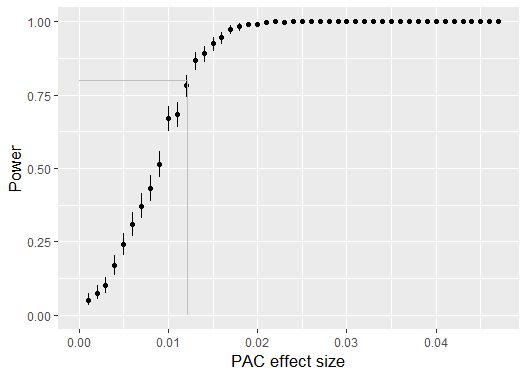


^1^ Green, P. & MacLeod, C. J. simr: an R package for power analysis of generalised linear mixed models by simulation. *Methods in Ecology and Evolution*, **7**, 493-498, doi: 10.1111/2041-210X.12504, (2016).
